# Mouse Models of Human Autoimmune Diseases: Essential Tools That Require the Proper Controls

**DOI:** 10.1371/journal.pbio.0020241

**Published:** 2004-08-17

**Authors:** Laurence Morel

## Abstract

What can we learn about human autoimmune disorders that have a genetic component -- such as systemic lupus erythematosus -- from mouse models?

Autoimmune diseases afflict a large segment of the population in Western countries. Many of them have been described, and rheumatoid arthritis, type I diabetes (also called insulin-dependent diabetes mellitus), multiple sclerosis, and systemic lupus erythematosus (SLE) are among the most common. Although tremendous progress has been made in disease management over the last decade, cures for these diseases have not yet been found. Consequently, a large research effort is sustained in this field. In addition, autoimmunity has intrigued basic immunologists since the early realization that the ability to discriminate self from non-self was at the core of the immune system's ability to protect an organism from pathogens while avoiding self-destruction. A failure of this mechanism results in autoimmune reactions that often lead to clinical disease. In spite of massive research efforts, the mechanisms by which autoimmune diseases develop are not clearly understood. Genetic predisposition as well as environmental triggers plays a role, but the identity of these factors has been largely elusive. The identification of the most common genetic and environmental factors that set off autoimmunity may lead to a better understanding of the ensuing pathogenesis, and offers the best hope for improved therapies, and ultimately, cures.

So far, animal models have proved the best way to probe the mechanisms of disease in general, and autoimmune diseases in particular. In the past few decades, the mouse has become the model of choice for experimental medicine, and the rat is following close behind. Starting in the early twentieth century at the Jackson Laboratory (Bar Harbor, Maine, United States), the production of inbred strains of mice and the systematic collection and characterization of naturally occurring mutants have created the building blocks on which much of the research using animal models is now based. Inbred strains are collections of genetically identical animals obtained through selective breeding. These strains have provided homogenous experimental groups, with interindividual variability reduced to environmental (and stochastic) factors. In addition, inbred strains have an assortment of distinct phenotypes that have then been exploited as models of human diseases.

Since the 1980s, techniques have been developed to manipulate the mouse genome. Specific genes now can be routinely over-expressed as transgenes, or eliminated by gene targeting, which creates a “knockout” (Smithies 1993; Wassarman and DePamphilis 1993). This approach, known as reverse genetics, has shown itself to be a powerful tool with which to evaluate the role of individual genes in various biological processes. The use of genetically engineered mouse models will likely play a major role in deciphering the function of a multitude of new genes revealed in the recently completed sequence of the mouse genome (Mouse Genome Sequencing Consortium 2002).

Interestingly, the percentage of mouse genes without any homolog currently detectable in the human genome (and vice versa) has been estimated to be less than 1%, a fact that strengthens the validity of mouse models of human diseases. However, one has to be careful in directly applying data obtained from animal models to human diseases. Most human autoimmune diseases show an extremely heterogeneous clinical presentation, which animal models present as simplified versions. A mouse model, as in any reductionist approach, is both inconvenient, because it provides only a partial representation of the real biological complexity underlying the human disease, and advantageous, because it is a more tractable tool with which to probe mechanistic issues. In addition, a number of differences exist between the human and rodent immune systems (Mestas and Hughes 2004). Since immune dysfunctions are at the root of autoimmune diseases, such differences may limit extrapolations from animal models to autoimmune patients.

Nonetheless, animal models are at the core of autoimmune research, and a large body of literature reflects the many advances brought by these models in terms of deciphering disease mechanisms. The relative lack of progress in certain human autoimmune diseases for which an animal model does not exist, such as neuropsychiatric lupus, corroborates the indispensable role played by animal models. Three basic types of animal models have been used in autoimmune research: spontaneous models, induced models, and genetically engineered models. The latter category has been further subdivided into transgenic and knockout strains (Table 1).

**Table 1:**
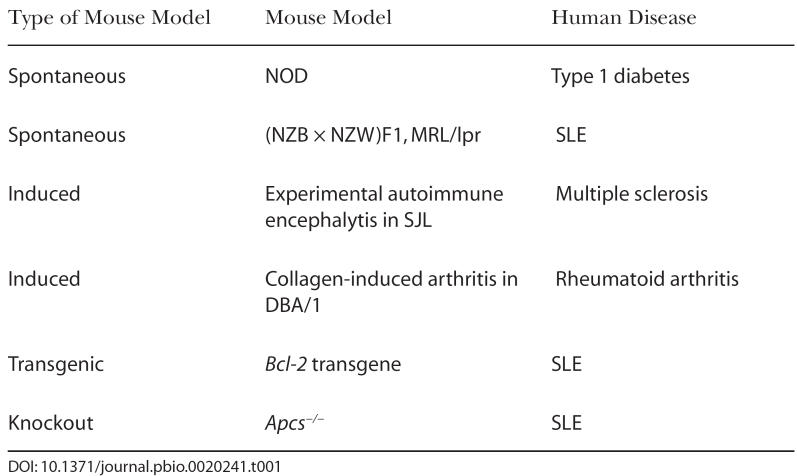
Examples of Common Mouse Models of Autoimmune Diseases

Spontaneous models were produced through fortuitous observations of clinical symptoms reminiscent of a given human autoimmune disease developing in a given mouse strain, or in crosses between mouse strains. This happened, for example, with the nonobese diabetic (NOD) mouse, which developed type 1 diabetes, and the hybrid between the New Zealand Black (NZB) and the New Zealand White (NZW) mouse, (NZB × NZW)F1, which developed a lupus-like disease.

Unfortunately, spontaneous models are not available for all human autoimmune diseases. Therefore, scientists have created induced models, often by exposing the animals to high doses of a suspected autoantigen at the same time as stimulating the immune system. Interestingly, marked differences exist between strains in their responses to these autoimmune inductions that reflect genetic variation associated with susceptibility to autoimmune diseases. For this reason, induced models have been limited to a small number of strains. For example, experimental autoimmune encephalomyelitis has been widely used as a model of multiple sclerosis. In this model, spinal cord homogenate or a protein derivative such as myelin basic protein is injected with a mixture of potent immunostimulants, most commonly in mice from the SJL strain. Another example of an induced model is collagen-induced arthritis, which has been used to study rheumatoid arthritis. In this model, type II collagen, a joint component, is injected also with immunostimulants, most commonly into mice from the DBA/1 strain.

Finally, the ability to turn specific genes on or off, in specific cell types and/or at specific times, has created a plethora of mouse models limited only by the immunologist's imagination. Genes suspected to play a role in the pathogenesis of various autoimmune diseases have been evaluated this way, and those studies have been very informative in mapping out functional pathways that are targeted in these diseases. However, these models have intrinsic problems that have become more apparent in the past few years, and require careful controls to avoid possible misinterpretation. These problems are a result of the fundamental way in which transgenic and knockout strains are produced. When a piece of DNA carrying the gene of interest is injected as a “transgene” into fertilized eggs, it integrates randomly into the genome, and in doing so, potentially modifies the expression of the gene it integrates into. Since all genetic studies have recognized that a large number of genes are involved in autoimmune disease susceptibility, the potential for a transgene to hit one of those susceptibility genes is not negligible. This potentially confounding factor is usually controlled by producing and comparing several independent transgenic lines.

More problematic is the interpretation of results obtained with knockout models. A gene is “knocked out” (KO) by homologous recombination of a disrupting piece of DNA within that gene. This genetic manipulation takes place in embryonic stem (ES) cells, which once mutated, are introduced into the inner cavity of a blastocyst (very early mouse embryo), creating chimeric embryos that are put back into female mice that carry the pregnancy to term. After multiple trials (and errors), ES cell lines from the 129 strain and blastocysts from the C57BL/6 (B6) strain have shown themselves to be superior in terms of efficiency and reliability. Consequently, this strain combination is at the origin of the overwhelming majority of knockout strains. Although the chimeric mice are usually backcrossed to B6 to dilute the contribution of the 129 genome, the knockout strains are always a mixture of the two genomes (Figure 1). Most importantly, a large region flanking the KO gene remains of 129 origin, unless extreme measures are taken to select for recombination between tightly linked markers. There have been sporadic reports of phenotypes initially attributed to deficiency in the expression of a given gene that disappeared with additional backcrosses to B6. The only possible interpretation was that these phenotypes were in fact due to 129 alleles that were replaced by B6 alleles with further backcrossing.[Fig pbio-0020241-g001]

**Figure 1 pbio-0020241-g001:**
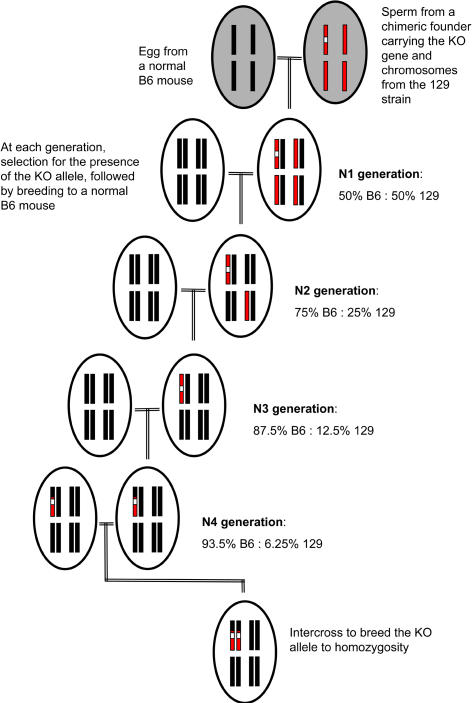
Breeding Strategy Usually Performed to Transfer a KO Allele from the 129 Genome to the C57BL/6 (B6) Genome For clarity, only 4 of the 20 pairs of the mouse chromosomes are represented by black (B6) or red (129) bars. The KO allele is shown by a white box. Chimeric males are obtained from the integration of ES cells from the 129 strain that have been engineered to carry the KO allele to B6 blastocysts. These males are then bred to normal B6 females, resulting in an N1 progeny that is made up of 50% B6 and 50% 129 genome. N1 mice are subsequently “backcrossed” to B6, and their N2 progeny is selected for the presence of the KO allele. The contribution of 129 genome among the N2 progeny is normally distributed around a mean of 25%. This process can be repeated (shown here to N4), resulting in an average reduction of the 129 genome to one half of what it was in the previous generation. At any point in the process, homozygosity for the KO allele, which is necessary to prevent expression of that gene, can be obtained by intercrossing heterozygous mice, shown here at N4.

The production of autoantibodies and mild antibody-related renal pathology, highly relevant to autoimmune diseases, especially SLE, has been reported independently in four mouse models using the (129 × B6) genetic background (Obara et al. 1979; Botto et al 1998; Bickerstaff et al. 1999; Santiago-Raber et al. 2001). It is generally accepted that genetic susceptibility to autoimmune diseases is conferred by multiple highly interactive genes that have small individual effects. In this context, the autoimmune phenotypes resulting from the combination of the 129 and B6 genomes may might therefore provide a primed background upon which the effects of deficiency in the target gene can be amplified. On the other hand, the autoimmune phenotype may be overwhelmingly contributed by the (129 × B6) genomic combination, with little if any effect of the deficiency of the KO gene.

In this issue of *PLoS Biology*, Marina Botto and her colleagues have tested this hypothesis by taking one of their knockout models for SLE, the serum amyloid P component deficient mouse (*Apcs* ^−/−^) (Bygrave et al. 2004). This group has published that *Apcs* ^−/−^ mice on a (129 × B6) genetic background develop a lupus-like disease, even after repeated backcrosses to B6 (Bickerstaff et al. 1999). Serum amyloid P component binds to debris generated from dying cells. Efficient removal of this debris has been shown to be critical to the prevention of the production of autoantibodies against intracellular material. *Apcs* was therefore an interesting candidate gene to evaluate. *Apcs*, however, is located in a region near the tip of mouse Chromosome 1 that is rich in SLE susceptibility loci and in genes that have been directly associated with SLE in humans (Wakeland et al. 1999). As mentioned above, the *Apcs* ^−/−^ mouse, although on a mostly B6 genomic background, has its entire *Apcs* flanking region replaced with 129 alleles. In a critical experiment, Botto and colleagues compared the autoimmune phenotypes of *Apcs* ^−/−^ mice to congenic mice (i.e., genetically identical, except for the gene of interest), carrying the same 129 region on Chromosome 1, but expressing *Apcs*. Amazingly, no difference was found between the two strains regarding the production of autoantibodies, clearly eliminating *Apcs* deficiency as a mechanism for this autoimmune process. *Apcs* deficiency was however associated with markedly increased renal damage, suggesting that this gene may be involved in preventing pathological consequences of autoantibody production.

It has been reported anecdotally that the most common outcome of a genetic knockout is a lupus-like disease. Botto and her colleagues may have identified the reason behind this somewhat surprising observation as a spurious consequence of the gene-targeting process. Gene targeting has been an invaluable tool in understanding the mechanisms of immunological diseases, and has still a very important role to play with increasingly sophisticated techniques of selective targeting. The immediate consequence of this work should be an increased scrutiny for appropriate controls, which may include congenic mice carrying the same 129 flanking region, but expressing the targeted gene (Figure 2).[Fig pbio-0020241-g001]

**Figure 2:**
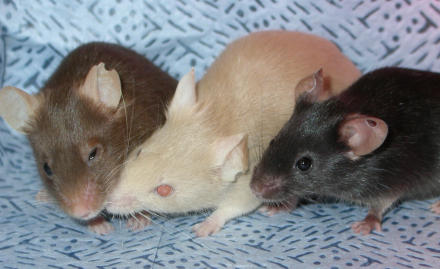
Congenic Strains (Left and Right) of a Lupus-Prone Mouse (Middle). Image courtesy of Jessica Merritto

The past few years have shown that genetic susceptibility to autoimmune diseases involves a large number of genes with small individual contributions. In spite of this great complexity, advances have been made, and a small but growing number of susceptibility genes have been identified (Morahan and Morel 2002). A common trait shared by successful studies has been the use of mouse models, either directly or indirectly. As the pace of genetic analysis increases in autoimmune diseases, and powerful tools have been created to navigate between the mouse and human genomes, the use of mouse models has been reaffirmed at multiple levels. Mouse models are used to discover new susceptibility genes that can then be assessed in patient populations, as well as to validate genes that have been directly identified in human genetic studies. Mouse models are also used to perform detailed functional and physiological analyses that cannot be conducted in humans. Finally, mouse models have been invaluable to screen disease-specific therapeutic agents.

Using mouse models has its pitfalls; many differences, both obvious and subtle, exist between mice and humans. Those differences are, however, outweighed by the power of the experimental system offered by the mouse. What the new study of Botto and colleagues reminds us is that the appropriate control is still crucial to meaningful data interpretation. Keeping that in mind, one can predict that many of the keys to human autoimmune diseases are still in the mouse room.

## Abbreviations

ES: embryonic stem
KO: knocked out
NOD: non-obese diabetic
NZB: New Zealand black
NZW: New Zealand white
SLE: systemic lupus erythematosus
